# Emerging probiotics: future therapeutics for human gut health

**DOI:** 10.1093/femsec/fiaf077

**Published:** 2025-07-25

**Authors:** Sylvia H Duncan, Carlos Sabater

**Affiliations:** Rowett Institute, University of Aberdeen, Foresterhill, Aberdeen, Scotland, AB25 2ZD, UK; Rowett Institute, University of Aberdeen, Foresterhill, Aberdeen, Scotland, AB25 2ZD, UK

**Keywords:** next-generation probiotics, *Faecalibacterium*, *Roseburia*, *Eubacterium*, *Ruminococcus*

## Abstract

The microbial communities that colonize the human large intestine can influence many aspects of health and *Bacillota* strains, in particular, have been proposed as next-generation probiotics. Of note are strains including fibre-degraders, butyrate producers, lactate producers and utilizers, and other beneficial metabolic activities that are important for health. To illustrate the potential applications of colonic bacteria to design novel prebiotic formulations, a comparative genomics analysis of 16 bacterial strains isolated from the human gut was performed. This analysis revealed a high number of carbohydrate-active enzymes (CAZymes) in the genome sequences of understudied *Lachnospiraceae* and *Oscillospiraceae* members including *Roseburia intestinalis* L1-82, *Roseburia faecis* M72/1, *Butyrivibrio fibrisolvens* 16-4, and *Ruminococcus bicirculans* 80/3, ranging from 32 to 56 CAZymes across different strains. These strains showed complementary enzymatic profiles covering a wide range of different activities required to degrade prebiotic substrates derived from vegetable sources such as arabino- and xylo-oligosaccharides and pectic-oligosaccharides. These metabolic differences highlight the potential of these strains to cross feed and to allow the design novel probiotic consortia for microbiota-oriented interventions that could target specific disease conditions. These bacterial strains are, however, strict anaerobes and therefore special measures may need to be put in place to overcome these limitations.

## Teaser

Fibre-degrading gut anaerobes including important butyrate producers, lactate producers and utilizers, have been proposed here as next-generation probiotics.

## Introduction

The human colon is colonized by a complex and dense variety of cells including fungi, bacteria, and viruses and each individual harbours several hundred different bacterial species. The three dominant phyla are the Bacteroidota (previously Bacteroidetes), *Bacillota* (previously Firmicutes), and Actinobacteriota (previously Actinobacteria) (Backhed et al. 2005, Gill et al. 2006, Rajilic-Stojanovic and de Vos 2014, Pasolli et al. 2019). The predominant genera within the Bacteroidota phyla are *Bacteroides* and *Prevotella* and the former is considered to predominate in China and USA populations with the latter predominating in Indian populations and these differences may be driven by differences in diets (Chung et al. 2020). Separately, the genus *Bifidobacterium* seems most prevalent in individuals living in The Netherlands (Wu et al. 2011, Sheng et al. 2023). The *Bifidobacterium* genus tends to be in rather low abundance in many individuals but is considered to have health benefits (Arboleya et al. 2016). Although certain variations appear to depend on countries of residence this most likely reflects differences in dietary intakes (Sheng et al. 2023, Ross et al. 2024). At the bacterial species level over half of the species were found in <1% of individuals (Flint et al. 2012), and certain species that were identified as being present in >90% of individuals include *Collinsella aerofaciens, Faecalibacterium prausnitzii, Anaerostipes hadrus, Blautia obeum, Eubacterium rectale, Eubacterium hallii*, and *Roseburia inulinivorans* (Duncan et al. 2002, 2006, Allen-Vercoe et al. 2012, Sheng et al. 2023). This may indicate that these species play a role in health maintenance, moreover these species alone account for around one-quarter of the total microbiota (Flint et al. 2012, Martin et al. 2013). In particular, *F. prausnitzii* appears to be present in a high proportion of individuals. These core microbial species listed above are all anaerobes that form a range of fermentation end products.

The composition of the gut microbiota may be markedly influenced by diet (Flint et al. 2012), other environmental factors such as pH, bile salt levels, and also antibiotics (Duncan et al. 2002, 2004). Other factors such as gut bacterial antimicrobials (Garcia-Gutierrez et al. 2019) including bacteriocins (Hatziioanou et al. 2013, Garcia-Gutierrez et al. 2019) and other antimicrobials may modulate the composition of the microbiota (Donia and Fischbach 2015). Many of these anaerobes that colonize the large intestine therefore have a role in energy harvest and generate many beneficial products for the host including short-chain fatty acids (SCFAs) and vitamins (Soto-Martin et al. 2020), in addition to antioxidant and anti-inflammatory metabolites thereby helping to promote health (Chung et al. 2017). These cells can interact with each other and also host cells to modulate a range of health aspects from metabolism of dietary substrates to impact on health and disease. To date studies have focussed more on bacterial species linked to disease and mechanisms to control the growth and activity of pathogens. Examples of the impact on disease include gastrointestinal disease such as *Clostridioides difficile* infections, in addition to links to inflammatory bowel disease, cardiovascular disease and a number of other conditions (Sehgal et al. 2021). Several other health conditions may be impacted by perturbations or an imbalance in the composition of each individual's gut microbiota sometimes referred to as microbial dysbiosis (Wu et al. 2013) and includes links to traveller's diarrhoea, inflammatory bowel disease including Crohn's disease (Manichanh et al. 2006), cardiovascular disease (Zaher et al. 2024), and also links across the gut brain axis (O'Mahony et al. 2015). As an example, an imbalance may include low abundance of the genus *Faecalibacterium* and proportionately high levels of *Escherichia coli* (Lopez-Siles et al. 2014). Microbial imbalance may result in the likelihood of infections from pathogens as reported in care homes where the prevalence is higher in the elderly compared to younger adults that may have perturbed microbiota (Duncan and Flint 2013, O'Toole 2024). There are a number of approaches that have been explored to help to retain and/or restore the balance of microbial species in the colon including faecal microbiota transplant (FMT), which has been shown to be effective in the treatment of *C. difficile* infections (Cammarota et al. 2015, Hanssen et al. 2021). This approach requires careful selection of donors, which is likely to be a challenge given the intervariability of microbial composition and although there is little evidence of side effects, such treatments can cause bacteraemia and hospitalization (Dailey et al. 2019, Zhou et al. 2021). The efficacy of FMT is likely to depend on the specific condition being treated and to some extent the composition of the donor's microbiota. There is clearly a need to refine this approach, however, the use of FMT (Dailey et al. 2019) should help pave the way to developing more targeted microbiome therapeutics such as targeted next-generation probiotic and therapeutic approaches to treat particular diseases. Moreover, both the use and overuse of antibiotics has led to high levels of antibiotic-resistant bacteria contributing to over 5 million deaths globally (Klein et al. 2024) and there are limited last line of antibiotics available in the clinic (Nang et al. 2021), therefore alternative treatments such as targeted next-generation probiotics or biotherapeutics is paramount (Al-Fakhrany and Elekhnawy 2024).

In particular, there are three functional groups of colonic bacteria that are of particular interest as next-generation probiotics and these are complex fibre degraders, butyrate producing bacteria, and lactate utilizing bacteria and these are considered below.

### Next-generation probiotics

The microbial communities that colonize the human large intestine can influence many aspects of health and in particular GI health. In the healthy state, these microbes contribute nutrients and energy to the host through the fermentation of complex dietary carbohydrates in the large intestine. Despite the intense research focus on understanding the composition of the gut microbiota, researcher have yet to definitively outline what comprises a healthy gut microbiota, although this has been covered in other papers (Van Hul et al. 2024).

**Figure 1.**
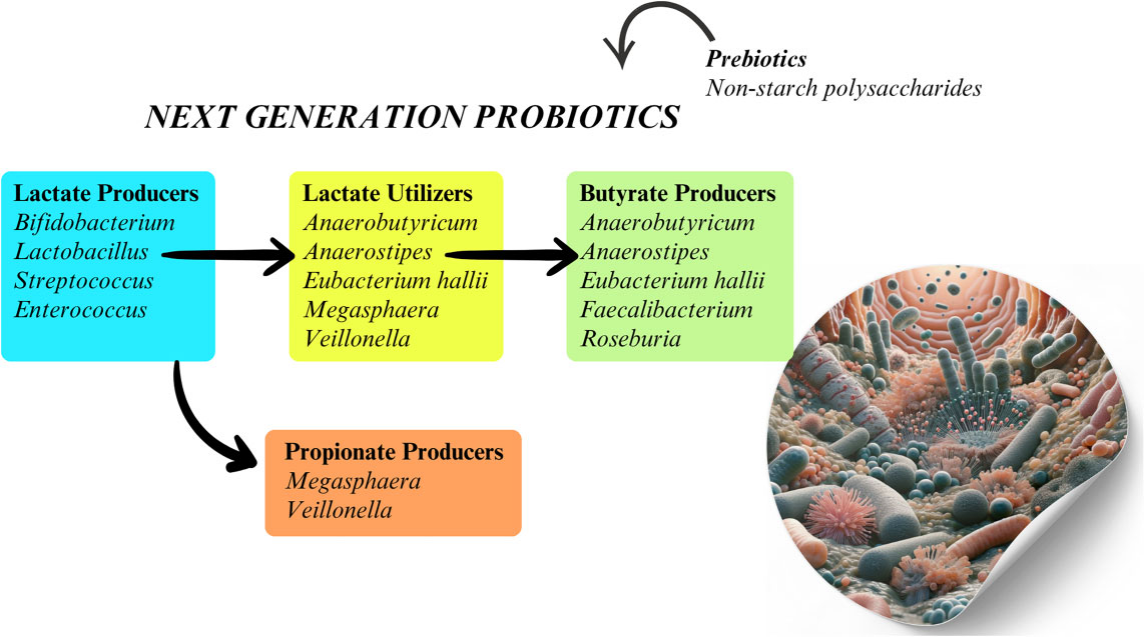
Relationships between lactate producers, lactate utilizers, and SCFAs producers (butyrate and propionate). Colour-coded sections and directional arrows highlight metabolic pathways and overlapping roles of bacterial genera.

The FAO/WHO partners have defined probiotics as living microbes that, when utilized in appropriate quantities, confer positive health benefits to their consumers. Most probiotic bacteria are Gram-positive and act through modulation and preservation of intestinal tract healthiness, such as *Lactobacillus* and *Bifidobacterium* species. In healthy adults, lactobacilli are mainly found in the small intestine and bifidobacteria, of which *B. adolescentis* is one commonly detected species in the colon of adults. This important bifid species is usually less than a few % of the total but varies depending on factors such as diet and age. It is therefore timely that alternative approaches to high usage of antibiotics and mechanisms to support colonic and other health conditions are developed (Al-Fakhrany and Elekhnawy 2024). Moreover, the establishment of next-generation probiotics may be enhanced with the addition of a targeted prebiotic, which should be a substrate that is likely to support the growth of the probiotic organism (Kandari et al. 2024). Fig. 1 provides a graphical representation of the process to obtain emerging prebiotics to support next-generation probiotics.

### Intestinal microbiota and metabolic function

It is also paramount to consider the key bacterial species that have a role in the metabolic outputs resulting from microbial fermentation of dietary macronutrients. This includes starch that is the carbohydrate in a typical diet whilst other nonstarch polysaccharides include cellulose, pectin, xylan, and mannan. These carbohydrates along with peptides that escape digestion by host enzymes are fermented by colonic bacteria to SCFAs, which can characteristically range from 50 to 150 mM. The three major SCFAs are acetate, propionate, and butyrate and the molar proportions of these can vary depending on dietary intakes (Flint et al. 2015). Acetate is mainly metabolized in the peripheral tissues, whilst propionate is gluconeogenic and metabolized in the liver and finally butyrate is a major energy source for the colonocytes (Louis and Flint 2017, Wang et al. 2020). Acetate is a fermentation product of most gut anaerobes, in differing proportions. By contrast propionate and butyrate are produced by subsets of gut microbes. Propionate is produced by human gut bacteria mainly using the succinate pathway or propanediol pathway with the latter using deoxy sugars such as fucose. *Bacteroides* and *Prevotella* species mostly generate acetate and propionate in the large intestine. Strains belonging to these genera mainly employing the succinate route for propionate formation. *Veillonella* species can convert lactate to propionate via the succinate route whilst *Megasphaera elsdenii* employs the acrylate route for propionate formation (Louis and Flint 2017).

### Butyrate producing *Bacillota* strains as next-generation probiotics

Given that butyrate is the major energy source for the colonocytes, which are the cells lining the colon, resulting in reducing inflammation and enhancing gut barrier function these bacterial strains are therefore strong candidates as next-generation probiotics (Boesmans et al. 2018).

Butyrate is produced by some of the most dominant bacterial species within the *Bacillota* phylum and includes *F. prausnitzii, E. rectale, Roseburia* species, *E. hallii*, and *Anaerostipes caccae*. These species all employ the butyryl CoA: acetate CoA transferase route for butyrate formation (Duncan et al. 2002, Vital et al. 2014, Louis and Flint 2017), which is the dominant route for butyrate formation in the human colon. There are other bacterial species however that use the butyrate kinase route, including *Coprococcus* species. Collectively these are amongst the most widely recognized and abundant species found in the large intestine across Western and non-Western countries (Flint et al. 2012, Sheng et al. 2023). In particular, *F. prausnitzii* (Duncan et al. 2002), which has been re-classified as three new species (Sakamoto et al. 2022) is one of the most abundant species present in the large intestine (Flint et al. 2012). Globally its abundance which is around 5%–8% of the total microbiota, is diminished in disease states. Re-establishing this bacterium is therefore likely to have key benefits (Sokol et al. 2008, Varela et al. 2013, Cao et al. 2014, Martin et al. 2014, 2015, 2023), particularly given its vital anti-inflammatory properties (Qiu et al. 2013, Miquel et al. 2015). This is at least partly driven through its butyrate producing capacity (Lenoir et al. 2020) in addition to the formation of a possible anti-inflammatory protein (Quevrain et al. 2016).

### Lactate producing and utilizing bacterial species from the human colon

Lactate is produced by a variety of bacterial species in the human colon (Duncan et al. 2004), and in particular is a main fermentation product of *Bifidobacterium* and *Lactobacillus* species whilst for other bacterial species this may be a minor fermentation product. Despite the numbers of microbes contributing to the pool of lactate in the colon, in the healthy colon lactate is usually found to be a minor product due to cross-feeding by lactate utilizers (Belenguer et al. 2006). This is due to a range of bacterial species found in the human colon possessing the ability to cross feed on lactate to form other products including butyrate and propionate (Duncan et al. 2004, Belenguer et al. 2006, Marquet et al. 2009). The fine balance of microbes can however be perturbed by changes in intestinal pH (Belenguer et al. 2007). The pH of the colon can markedly impact on the composition of colonic microbiota (Duncan et al. 2009) and as the pH of colonic contents decrease, lactate levels may increase, resulting in poor health outcomes such as neurocardiac toxicity (Chan et al. 1994).

Babies tend to have a high proportion of bifidobacteria, which will form lactate, along with acetate, as a fermentation end product. The importance of gut bacteria to metabolize lactate in the infant intestinal tract is likely to be high, as lactate accumulation has been associated with colic in infants (Pham et al. 2017). The lactate utilizing species *A. caccae* may also have a protective role against the development of food allergy (Hesser et al. 2024). Lactate accumulation can result in surgical removal of portions of the small and large intestine in humans (Kowlgi and Chhabra 2015), and with gut disorders such as the IBD, Crohn's disease, and ulcerative colitis (Hove et al. 1994). There is also evidence to suggest that lactate accumulation also correlates with disease severity in IBD. Vernia et al. (1988), e.g. showed that ulcerative colitis patients with severe symptoms had elevated faecal lactate concentrations along with reduced concentrations of the SCFA, butyrate.

Lactate accumulation can have a major impact on destabilizing the intestinal microbiota (Louis et al. 2022). In particular, lactic acid accumulation can also be used as a carbon source for pathogens including *Salmonella* that can oxidize lactate to carbon dioxide and water (Gillis et al. 2019). Other detrimental bacterial species include *Desulfovibrio* species, and the most abundant species in the human colon is *Desulfovibrio piger*, which can metabolize lactate to acetate and hydrogen sulphide through dissimilatory sulphate reduction (Marquet et al. 2009). Lactate serves as the primary electron donor and is oxidized to acetate, carbon dioxide, and sulphate acts as the terminal electron acceptor. The reduced product, hydrogen sulphide is genotoxic (Attene-Ramos et al. 2010).

The lactate-utilizing bacteria that can help to re-stabilize the colonic ecosystem are therefore crucial in preventing acidosis and these bacteria are likely to be a key target for new biotherapeutics. This includes bacterial species that can utilize lactate to form propionate, including *Coprococcus catus* and *M. elsdenii* employing the acrylate pathway (Louis et al. 2022). In this regard, cross-feeding interactions between lactate-producing and lactate-utilizing bacteria include lactate cross-feeding between *Bifidobacterium* and *Megasphaera* species. *Bifidobacterium* hydrolyses xylan structures to catabolize xylose leading to lactate formation. Then, *Megasphaera* species utilize lactate to generate butyrate as its end metabolic product (Zhao et al. 2024). Other bacterial species that utilize lactate (along with acetate) include *Anaerobutyricum* and *Anaerostipes* species to generate butyrate. Specific representatives of all these bacterial species that can use lactate, as a carbon and energy source, should be given especially high consideration for development of next-generation probiotics.

Although there have been rather few trials that have been performed to demonstrate the efficacy of lactate-utilizing bacteria as probiotics in humans, a small-scale human study did show that orally dosed *Anaerobutyricum soehngenii* improved peripheral insulin sensitivity (Gilijamse et al. 2020), which was accompanied by altered microbiota composition and importantly was judged to be safe. In a separate study, duodenal infusion of *A. soehngenii* in individuals with metabolic syndrome also showed success (Koopen et al. 2022).

### Key fibre degrading colonic anaerobes

Other than alternative energy sources for colonic bacteria, there is much interest in the role of dietary fibre in promoting health. Fibre rich foods are fruits, vegetables, whole grains, and legumes and diets high in dietary fibre can prevent and relieve constipation, influence control appetite to benefit weight management and stabilize blood sugar levels (Scott et al. 2008). Fibre can also lower cholesterol, which in turn reduces the risk of heart disease (Duncan et al. 2023). It also helps maintain healthy blood pressure levels. High-fibre diets may lower risk of developing certain types of cancer, such as colorectal cancer (Scott et al. 2008, Duncan et al. 2023).

The daily recommended intake of 30 g per day fibre is considered to improve general health including heart health, type-2-diabetes, and colorectal cancer. This is largely driven by the impact of dietary fibre that largely escapes digestion by host enzymes reaching the large intestine and being fermented by key members of the gut anaerobes (Duncan et al. 2023). Other species that have a role complex carbohydrate utilization, include *Ruminococcus bromii*, which is a keystone species for resistant starch metabolism. It has been shown that the low abundance of this microbe results in incomplete metabolism of resistant starch (Ze et al. 2012). The main cellulolytic bacterium for cellulose degradation is *Ruminocoocus champanellensis* (Morais et al. 2016). Separately, *Eubacterium eligens* that metabolizes pectins possesses potent anti-inflammatory activity (Chung et al. 2017). Other *Bacillota* including *E. rectale* and *Roseburia* species have a key role in metabolizing complex carbohydrates such as arabinoxylans (Duncan et al. 2016) and are major butyrate producers and this is a metabolite of particular interest to promote and maintain health.

### Prebiotic substrates to support next-generation probiotics

Considerable progress has been achieved in defining the dominant microbial species and their metabolism within the large intestine. It is evident that diet and/or prebiotics has a major influence on microbial composition. These changes can occur within a few days of diet shift, which is encouraging with respect to influencing health (David et al. 2014). It is, however, crucial to determine the possibilities of linking new and/or next-generation probiotics with their optimal prebiotic substrates in order to increase their ability to survive and persist in the human colon.

Next-generation prebiotics are being designed and developed to enhance the growth and activities of beneficial gut microbes to improve overall health. Fruit and vegetable by-products are a source of promising prebiotics including arabino- and xylo-oligosaccharides (AXOS and XOS), galacto-oligosaccharides, manno-oligosaccharides, and pectic-oligosaccharides (POS). For example, POS can be extracted from pectin through enzymatic, chemical, or physical treatments, resulting in various compounds with different structures and properties (Wongkaew et al. 2022). The methodology exerts a great influence on the structural features and fermentative properties of these compounds (Sabater et al. 2021). It has been proposed that the beneficial effects of complex prebiotic structures are the result of cross-feeding mechanisms among cooperative gut commensals. Nutrient cross-feeding is an important metabolic interaction mechanism of bacterial groups inhabiting the human colon. Gut commensals possess an elaborate array of specific enzyme activities and intermediate carbohydrate breakdown products and certain fermentation products serve as carbon and energy sources for cross-feeding bacteria (Rios-Covian et al. 2015). Health promoting effects of prebiotic substrates including SCFAs and the inhibition of potential pathogenic bacteria are sometimes the result of cross-feeding mechanisms among cooperative bacteria in the human gut. It should be noted that some complex fibre structures can only be directly accessible to a limited number of gut commensals, yet they might provide benefits to various microbial populations within the community through synergistic metabolic interactions (Sabater et al. 2021). This metabolic complementarity enhances the stability and resilience of the microbiota resulting in health benefits for the host.

One role for these prebiotic substrates is that they can favour bifidobacteria that can benefit health directly. Alternatively, lactate may be used by cross-feeders such as certain butyrate producers or alternatively fuel butyrate-producing bacteria directly. In this regard, the bifidogenic and butyrogenic effect of AXOS is the result of cross-feeding mechanisms between bifidobacteria and eubacteria. For example, *E. rectale* can release xylose monomers from AXOS and promote the growth of *Bifidobacterium longum*. Similarly, *B. longum* produces acetate, which is further used by *E. rectale* to produce butyrate (Moens et al. 2016, Riviere et al. 2018). These metabolic interactions suggest a high specialization of gut microbiota to metabolize specific regions of complex poly- and oligosaccharide chains. Recent advances in next-generation sequencing and bioinformatics methods allow studying the presence of different carbohydrate-active enzymes (CAZymes) in the genome sequences of gut commensals.

**Figure 2.**
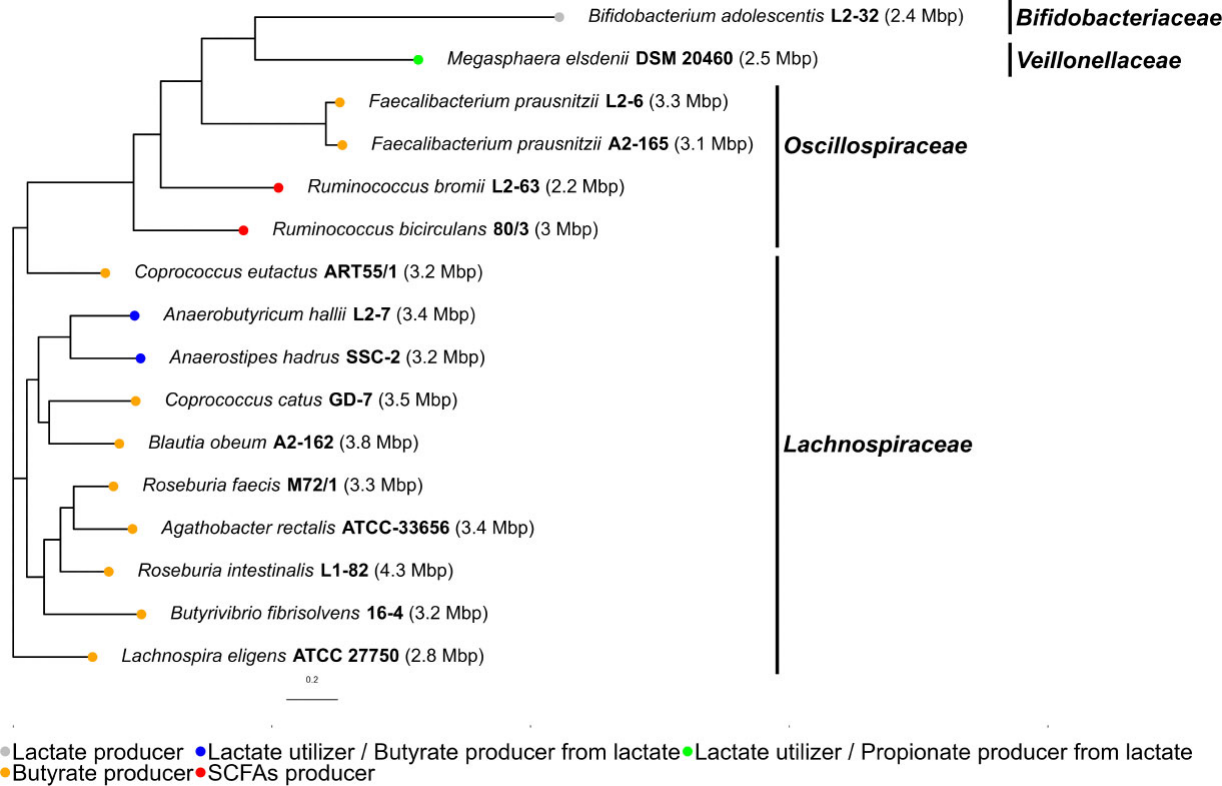
Phylogenomic tree of emerging probiotic strains from *Bifidobacteriaceae, Veillonellaceae, Oscillospiraceae*, and *Lachnospiraceae* families. Colour codes highlight the metabolic capabilities of each strain.

**Figure 3.**
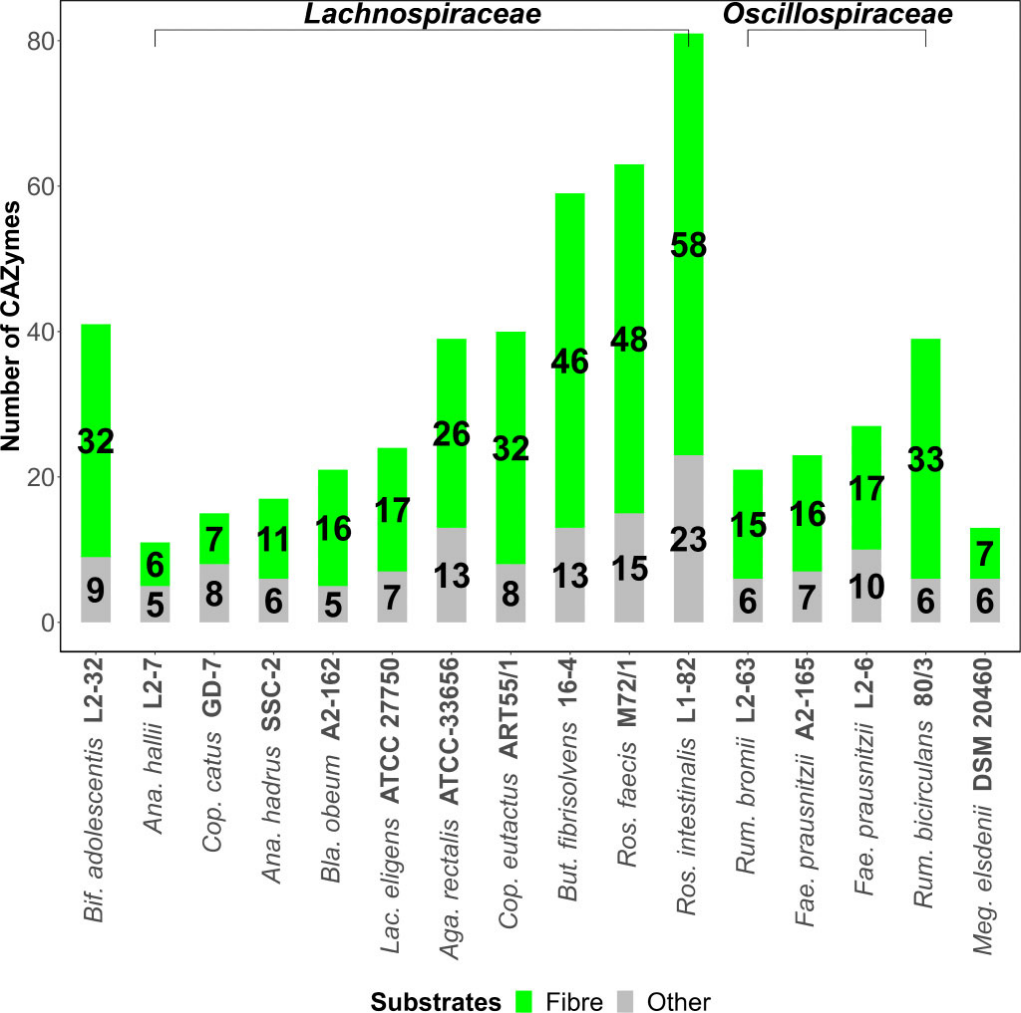
Total number of CAZymes acting on fibre (Fibre, defined as the sum of pectin, xylan/arabinoxylan, mannan, glucan/xyloglucan, fructan and lignocellulosic materials) and other carbohydrate substrates (Other) determined in the genome sequences of emerging probiotic strains.

To illustrate the potential applications of genome mining to design novel prebiotic formulations that are selectively metabolized by gut bacteria, a comparative genomics study of 16 next-generation probiotics was carried out (Figs. 2 and 3). Genome sequences of these bacteria were retrieved from the NCBI Assembly database. A phylogenomic tree was first computed using UBCG pipeline (Na et al. 2018). As expected, members of *Lachnospiraceae, Oscillospiraceae, Veillonellaceae*, and *Bifidobacteriaceae* families were clustered in different clades, while multiple species corresponding to the same genus (*Roseburia, Ruminococcus*, and *Faecalibacterium*) formed an independent subclade (Fig. 2). In addition, *Roseburia* species, *Butyrivibrio fibrisolvens* 16-4, and *Agathobacter rectalis* (previously *E. rectale*) ATCC-33656 were closely related according to the phylogenomic tree.

Then, complete CAZymes profiles found in the genome sequences of these probiotics were determined (Fig. 3). For this purpose, 'run_dbcan' software developed previously (Zhang et al. 2018), which maps the samples against the CAZy database [http://www.cazy.org (3 February 2025, date last accessed)], was selected to annotate different CAZymes families. To ensure the quality of the data generated, only glycosidase domains showing coverage values higher than 0.8 were chosen. The total number of CAZymes acting on fibre structures was first investigated (Fig. 3). In general, microbial strains showing the highest genome sizes also showed the widest range of CAZymes. *Lachnospiraceae* members showing the highest number of fibre-degrading domains were *Roseburia intestinalis* L1–82, *Roseburia faecis* M72/1, and *B. fibrisolvens* 16-4 (*n* = 46–58 CAZymes), while *Ruminococcus bicirculans* 80/3 showed the highest number of CAZymes acting on fibre (*n* = 33 CAZymes) among *Oscillospiraceae* members. In addition, *Bifidobacterium adolescentis* L2-32, one representative Actinobacteriota included in this study, showed also a high number of fibre-degrading domains (*n* = 32 CAZymes).

**Figure 4.**
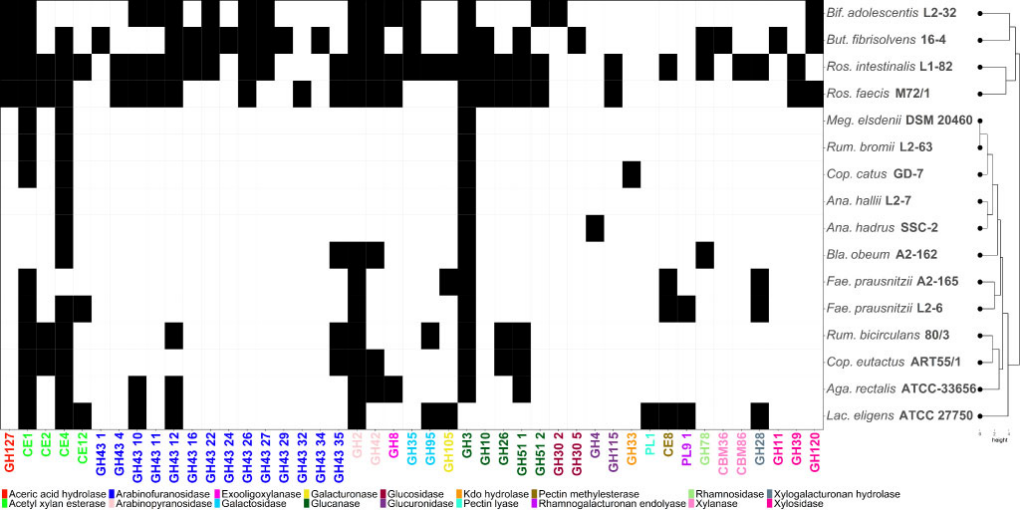
Heatmap showing the distribution of CAZymes acting on POS, AXOS, and XOS structures. Codes corresponding to the CAZy family of each enzyme have been assigned. Black and white cells indicate presence/absence of each CAZyme, respectively.

Once complete CAZymes profiles were annotated, the distribution of specific metabolic activities acting on two families emerging prebiotics, POS and AXOS/XOS, were compared through hierarchical clustering using R (v.4.2.3) basic function 'hclust' (Fig. 4 and 5). With regard to POS metabolism, four strains showed the widest range of enzymes acting on POS (Fig. 4): *Lachnospira eligens* ATCC 27750, *R. intestinalis* L1-82, and *F. prausnitzii* strains L2-6 and A2-165. These enzymatic activities involve polygalacturonases and xylogalacturonases that metabolize both linear oligogalacturonide structures as well as rhamnosidases, galactosidases, and arabinopyranosidases acting on ramified chains derived from rhamnogalacturonan rich in neutral sugars. Pectin methyl- and acetylesterases acting on substituted galacturonic acid residues were also determined in these strains. On the other hand, *B. fibrisolvens* 16-4 showed a wide range of CAZymes acting on ramified chains. Similarly, *B. obeum* A2-162, *Coprococcus etactus* ART55/1, *R. faecis* M72/1, *R. bicirculans* 80/3, and *Agathobacter rectalis* (previously *E. rectale*) ATCC-33656 showed enzyme activities acting on POS derived from rhamnogalacturonan and no CAZymes acting on oligogalacturonides. In contrast, *A. hadrus* SSC-2 showed only galacturonase domains and may not completely metabolize POS derived from ramified pectin chains. No POS-degrading domains could be annotated in the genome sequences of *Anaerobutyricum hallii* L2-7, *R. bromii* L2-63, and *M. elsdenii* DSM 20460. These results suggest a metabolic complementarity between different groups of bacteria with complementary activities in order to metabolize POS derived from linear and ramified pectin regions.

The study of CAZymes acting on XOS revealed a high number of enzyme domains in the genome sequences of *B. fibrisolvens* 16-4, *R. intestinalis* L1-82, *R. faecis* M72/1, and *B. adolescentis* L2-32 (Fig. 4). These enzymes involved glucanases and other CAZymes involved in the release of arabinose and xylan monomers. The rest of strains under study showed glucanase and acetyl xylan esterase domains and may require synergistic interactions with other strains showing a variety of arabinases and xylanases in order to fully metabolize XOS and AXOS structures.

### Other promising human gut probiotics

Here we focussed on highlighting important human gut bacteria that carry out important functional roles for the host. The emphasis therefore is on complex carbohydrate metabolism and butyrate formation. Importantly, the other functional group of interest are the lactate utilizing species. Other than the functional groups commented on above, there are clearly, however, several other bacterial species of note, including *Akkermansia mucinophila*. This bacterium inhabits the mucous layer of the large intestine and metabolizes host mucin and glycoproteins as an energy source. This in turn increases mucin production, which strengthens the intestinal barrier and reducing permeability (Cunningham et al 2021).

Another bacterial species gaining interest is *Coprococcus eutacus*, as its abundance is correlated with several neurological disorders. The mechanism for this is not well understood but may be linked to particular metabolites formed by *C. eutactus*, such as indole-propionic acid, which is formed from the metabolism of tryptophan (Menni et al. 2019). For example, low levels of *C. eutactus* is correlated with depression (Valles-Colomer et al. 2019) and this bacterium has been recognized as a valuable target for probiotic development or to be promoted through prebiotic approaches (Notting et al. 2023). As we gain a deeper understanding on the role of gut microbes in promoting health other candidates will also be contenders for development as live biotherapeutics.

## Discussion

Samples collected from the human large intestine are likely to be a valuable source of beneficial microbes with health protective properties. There are several studies that have identified the most abundant species present in the large intestine and that may play a health protective role revealing their potential as next-generation probiotics (Tiwari et al. 2024). In particular, these mainly include bacterial species belonging to the *Bacillota* phylum and that in particular produce butyrate as their major fermentation end product. This is important given that butyrate has a number of beneficial properties and is formed by a number of colonic bacterial species including strains belonging to the *Faecalibacterium, Roseburia*, and *Eubacterium* genera.

In addition to efficacy testing, there are, however, other important barriers to overcome before obligately anaerobic lactate-utilizing bacteria could be widely adopted as novel probiotics. Most species are highly oxygen sensitive, meaning novel delivery methods may need to be developed. Moreover, unlike traditional probiotics, such as lactobacilli and bifidobacteria, which are generally regarded as safe, comparatively much less is known about most anaerobic lactate-utilizing bacteria. Assessing the safety of any new probiotic is therefore paramount (Sanders et al. 2010) and it is essential that each strain is thoroughly screened for undesirable traits such as antibiotic resistance, virulence factors and toxin formation. Nonetheless, many species, particularly those that can convert lactate into the beneficial SCFAs including butyrate, are undoubtedly appealing candidates for further development (Van Immerseel et al. 2010) and there are studies that report on the safety and tolerance of butyrate producers, in trials, including *Butryicicoccus pullicaecorum* (Boesmans et al. 2018).

Comparative genomics of human gut bacteria allows identifying metabolic activities involved in the degradation of different types of fibre and beneficial metabolite production. In this regard, different bioinformatics methods can be used to gain a better understanding of the structure-activity relationships of prebiotics derived from fruit and vegetable sources. This information could be of great interest to tailor novel prebiotic formulations that selectively modulate understudied gut anaerobes including *Lachnospiraceae* and *Oscillospiraceae* families. The study of CAZymes profiles of these bacteria and their metabolic complementarity could be used to design novel probiotic consortia. Previous studies highlight the potential applications of these emerging probiotics to develop microbiota-oriented interventions that could target specific disease conditions (Lordan et al. 2020). These computational predictions must be validated in the laboratory and supported by experimental evidence.

FMT is also attracting interest to treat certain disease conditions, particularly bowel disorders and FMT, which is the transfer of faecal bacteria from a healthy donor to another individual (Taur et al. 2018, Ooijevaar et al. 2019). For example, Paramsothy et al. (2019) reported that the use of FMT in ulcerative colitis patients resulted in remission when there was enrichment of several species including two lactate-utilizing bacteria, namely *A. hallii* and *A. hadrus*, when compared to patients that did not achieve remission. These findings should help to underpin the importance of these two species for human health promotion and support their potential as next-generation probiotics.

## Conclusions

Comparative genomic analysis of gut isolates revealed that there are several bacterial species that are habitually present in the human colon that are likely to offer excellent opportunities to be developed as next-generation probiotics. In particular this includes key fibre degraders, butyrate producers, and lactate utilizers. These understudied emerging probiotics are mostly *Lachnospiraceae* and *Oscillospiraceae* members, showing complementary enzyme profiles involved in the degradation of prebiotic substrates derived from vegetables such as POS, AXOS, and XOS. This metabolic complementarity could be useful to design novel probiotic consortia for microbiota-oriented interventions. The challenge working with many of these bacterial strains is their stability and resilience as they are strict anaerobes and are considered to only survive for short periods in air and therefore how these can be best preserved for delivery as probiotics. A feasible option may be to microencapsulate anaerobic probiotics using matrices such as sodium alginate, which would protect cells (Phùng et al. 2024). Given that there is a particular challenge when working with single strains, a well characterized mini consortia may offer a better option. A bacterial consortium may benefit from a number of factors including functional complementarity such as cross feeding of complex carbohydrates giving broader substrate utilization and also provide essential growth factors, such as vitamins. Syntrophic interactions may also occur with one species consuming the by-products of another as is the case with lactate producing and utilizing species. Certain bacterial species may also survive exposure to oxygen better than others whilst certain bacterial species can use oxygen as a terminal electron acceptor.

There are clearly a number of exciting avenues being pursued to promote gut health and that may offer alternatives and other treatments to antibiotics and to help reduce the spread of antibiotic-resistance bacteria.
